# Violence against children with disabilities in a rural population of Upper Egypt: a community-based study

**DOI:** 10.1186/s42506-025-00200-3

**Published:** 2025-11-20

**Authors:** Wafaa A. Ali, Ahmed M. Gad-Allah, Seham A. Abokresha, Abdel Rahman Z. Abdel Rahman, Asmaa M. khalaf

**Affiliations:** 1 Forensic Medicine and Clinical Toxicology Department, Faculty of Medicine, Sohag University, Sohag, Egypt; 2 Forensic Medicine and Clinical Toxicology Department, Faculty of Medicine, Al- Azhar University (Assiut Branch), Assiut, Egypt; 3 Public Health and Community Medicine department, Faculty of Medicine, Sohag University, Sohag, Egypt

**Keywords:** Children’s disabilities, Rural, Risk factors, Upper Egypt, Violence

## Abstract

**Background:**

Children with disabilities face heightened violence risks, particularly in resource-limited settings. Despite global evidence, data on violence against children with disabilities in rural Upper Egypt remain scarce. This study aimed to determine the prevalence, types, and family-related risk factors of violence against children with disabilities in this region.

**Methods:**

A community-based cross-sectional study was conducted (December 2023–March 2024) in two rural Upper Egypt villages. Using a multistage random sampling, 213 young adults (aged 18–24 years) with disabilities were included. Data were collected using validated structured interviews (ISPCAN tool) and focus group discussions.

**Results:**

The sample comprised 54% males and 46% females. Around 50% primarily had intellectual, and 33.8% had physical disabilities. Emotional violence was nearly universal (93.1 in males, 100% in females), followed by physical (73.6–78.6%) and sexual violence (21.1–26.4%). Females experienced higher violence rates (50% vs. 9.6% males, *p* < 0.001). Parental drug abuse (22.6% males vs. 10.2% females, *p* = 0.02) and low socioeconomic status (59.1% vs. 43.9%, *p* = 0.03) were significant risk factors. Intellectual disabilities correlated with elevated sexual violence Perpetrators included parents (35.4–41.8%) and unrelated individuals (44.3–60%).

**Conclusions:**

Systemic violence against children with disabilities in rural Upper Egypt was pervasive, driven by poverty, low parental education, and gender disparities. Interventions must address familial and community-level factors, including economic support, parental education, and stricter enforcement of child protection laws.

## Introduction

It has been widely accepted that children, due to their age and developmental stage, are especially vulnerable to abuse and maltreatment. The risk of exposure to victimisation is substantially increased when a child experiences impairment and disability [1]. A systematic review, in 2016 revealed that disabled children have suffering from violence four times than their normal peers [2]. This vulnerability varies by disability type, with conditions such as brain injury, speech disability, and physical disability requiring particular attention [3].

Non-sexual child maltreatment has been causally linked to various mental disorders, drug use, suicide attempts, and risky sexual behaviors, emphasizing the serious long-term consequences [4]. Children with disabilities already experience increased risk of mental health difficulties but are often overlooked when designing school-based interventions [5]. The relationship between disability and psychopathology is complex, with much of the elevated risk stemming from increased exposure to psychosocial disadvantage [6].

In Egypt specifically, nearly half of the children have been subjected to severe violent disciplinary practices at home [7]. Bayesian geospatial modeling reveals widespread child labor across Egypt, with poverty, neglect, and exposure to violence identified as key drivers, particularly affecting children with disabilities [8]. Studies of child homicide in Egypt show perpetrators are predominantly male, especially fathers, with risk factors including low paternal education, separated couples, and rural residency [9].

Despite Egypt's Child Law mandating protection for children from violence, stronger enforcement is needed to eliminate violent disciplinary methods [7]. Health administrations must focus efforts on less educated, rural, and unstable families, evaluating intrafamilial violence risk factors and offering psychological support to potential perpetrators to protect vulnerable children with disabilities [9].

To the best of our knowledge, there is a lack of available data regarding violence against children with disabilities in rural populations of Upper Egypt, as assessed through community-based surveys. Our study aimed to determine the prevalence, types, magnitude, and frequency of violence against children with disabilities in a rural population of Upper Egypt, as well as to identify important family-related risk factors contributing to such violence.

## Methods

### Study designs and setting

A community-based cross-sectional study was conducted from December 2023 to March 2024 on 213 young adults aged 18–24 years with disabilities who recalled their childhood experience of violence.. Participants were residing in two rural areas of Upper Egypt: El Shikh Shiple and Bnawit villages, located in El Mragha district, Sohag governorate, Egypt. The study locations were selected through a multistage random sampling technique. Initially, Sohag governorate, which comprises 12 districts, was targeted, from which El Mragha district was randomly selected in the first stage. During the second stage, two villages—El Shikh Shiple and Bnawit—were randomly chosen from the 23 villages within the district. According to the Central Agency for Public Mobilization and Statistics, [10] these villages had population sizes exceeding 15,187 and 21,907 inhabitants, respectively.

Ethical approval for the study was obtained from the Medical Research Ethics Committee of the Faculty of Medicine at Sohag University, in accordance with institutional standard operating procedures and guidelines. Soh-Med-15–10-7PD. Informed written consent was obtained from all participants (young adults with disabilities and their responsible caregivers) prior to inclusion in the study.

### Sample size

The sample size calculation was performed using EpI-Info 2002 software statistical package designed by the World Health Organization (WHO) and by Centers for Disease Control and Prevention (CDC).

The sample size was calculated based on the following considerations: 95% confidence level and 88.8% incidence of physical violence in disabled children according to a previous study [11] ± 4.5% confidence limit. Twenty-four cases were added to overcome possible dropout. Therefore, we recruited 213 cases. The total number of eligible individuals identified during community screening was 274. Out of these:

Two hundred thirteen participants agreed and were included in the study, 28 individuals did not meet the inclusion criteria (no official documentation of disability), and 33 young adults were invited but declined participation, mainly due to lack of interest or unavailability.

### Selection criteria

Participants with disabilities included were previously diagnosed with physical impairments (those interfering with activities of daily living), cognitive disabilities (intellectual disorders substantially limiting one or more major life functions), and sensory impairments (hearing and visual). The inclusion of diverse disability types enabled comprehensive representation of the target population.

The retrospective nature of the study allowed for the collection of data on experiences of violence up to the age of 17 years, providing insights into childhood experiences while mitigating ethical concerns related to interviewing minors about sensitive topics.

Data collection was executed in two distinct phases. In the first phase, a comprehensive list of households with disabled individuals was compiled from multiple sources, including the local health office, the local social unit of the Egyptian Ministry of Social Solidarity, and non-governmental organizations dedicated to disability support. Rigorous verification of disability status was performed by contacting householders and requesting documentation of impairment. This documentation included hospital records, physician statements, or investigation reports. Households unable to provide such documentation were excluded from the sampling frame to maintain the integrity of the study population.

In the second phase, systematic random sampling was employed to recruit participants while ensuring appropriate representation across gender, socioeconomic background, and disability type. The sampling process reflected the prevalence of disabilities within the same age group in the general population. The initial household was randomly selected, and subsequently, every second household was visited following a randomly determined direction. To minimize non-response bias, houses that were unoccupied during the initial visit were revisited on three consecutive days before being excluded from the study. This constituted the third stage of the multistage sampling procedure.

### Data collection

Six trained data collectors conducted retrospective interviews with participants regarding experiences of violence, including sexual violence, that occurred before reaching 18 years of age. The data collectors received extensive training to handle the subject matter sensitively and to maintain ethical standards throughout the interview process. Participants (young adults with disabilities and their responsible caregivers) were thoroughly informed about the nature and objectives of the research, their right to decline participation, and their freedom to discontinue the interview at any time. Informed consent was obtained from all participants, and anonymity and confidentiality were strictly maintained throughout the study. All interviews were conducted in private settings to ensure participant comfort and confidentiality.

Two primary data collection tools were utilized:

1. A structured interview questionnaire (The retrospective version) adopted from the International Society for the Prevention of Child Abuse and Neglect (ICAST-R) = ISPCAN Child Abuse Screening Tool – Retrospective version [12]. This instrument was designed to generate predominantly quantitative data concerning the cause, nature, magnitude, and impact of violence experienced by participants. The questionnaire incorporated qualitative and open-ended questions to provide contextual depth and allow participants to detail their personal experiences and observations. The questionnaire was structured into three major sections addressing physical, emotional, and sexual violence.
  Structure & Content

Core items: 44 main questions on potentially abusive acts were collected from previous studies. Each endorsed item has follow-up questions: Frequency → how often it happened. Perpetrator → who did it (parent, relative, teacher, etc.). Timing → at what age(s) it happened. It covers main maltreatment domains: Physical abuse (12 question), Emotional/psychological abuse (23 question), and Sexual abuse (9 question).

It was first translated into Arabic and then back-translated into English to ensure accuracy.

A pilot study was conducted on 50 participants, who were later included in the main study. Internal consistency and reliability were assessed using Cronbach’s alpha coefficient, which indicated an acceptable level of reliability for all subscales: Internal consistency (Cronbach’s alpha): Physical abuse ≈ 0.60, Psychological ≈ 0.71 and Sexual ≈ 0.76

(2) Semi-structured focus group discussions were conducted with relevant stakeholders, including local social specialists from governmental schools, representatives from the Egyptian Ministry of Social Solidarity, and personnel from non-governmental organizations supporting individuals with disabilities. These discussions were instrumental in contextualizing the social and economic environment surrounding children with disabilities and facilitated the exploration of complex and open-ended questions related to the research objectives.

### Statistical analysis

Statistical analysis was done by SPSS v26 (IBM Inc., Chicago, IL, USA). Qualitative variables were presented as frequency and percentage and were analyzed using the Chi-square test. A two-tailed *P* value ≤ 0.05 was considered statistically significant.

## Results

The study included 213 young adults with disabilities who recalled their childhood experience of violence (54% male, 46% female) from rural Upper Egypt. Most families had 4–6 children (62%), and over half of the participants were firstborn (51.2%). Socioeconomic status was predominantly low (64.8%), and caregivers were primarily both parents (67.6%), and the remainder were cared for by a single parent or other family members. Intellectual disabilities were the most common (50.7%), followed by physical (33.8%), auditory (8.9%), and visual (6.6%) disabilities. Education levels were notably low, with 51.6% of caregivers being illiterate and only 3.3% attaining a university education. These demographics highlight the intersection of poverty, limited education, and care-giving structures in this population (Table 1).

**Table 1:** Socio-demographic characteristics of the studied young adults with disabilities, Rural Upper Egypt, 2023–2024

Characteristics	*N* = 213 N (%)
**Gender**	
Male	115 (54)
Female	98 (46)
**Number of children in the family**	
1–3	48 (22.5)
4–6	132 (62)
> 6	33 (15.5)
**Birth order**	
First	109 (51.2)
Second	49 (23)
Third	25 (11.7)
Fourth	19 (8.9)
Fifth or more	11 (5.2)
**Socioeconomic level**	
Low	138 (64.8)
Middle	46 (21.6)
High	29 (13.6)
**Level of education**	
Illiterate	110 (51.6)
Primary	47 (22.1)
Preparatory	32 (15)
Secondary	17 (8)
University	7 (3.3)
**Type of caregiver**	
One parent	26 (12.2)
Both parents	144 (67.6)
Sister	6 (2.8)
Brother	18 (8.5)
Other	19 (8.9)
**Type of disability**	
Physical	72 (33.8)
Intellectual	108 (50.7)
Auditory	19 (8.9)
Visual	14 (6.6)

Significant risk factors for violence included female gender (50% vs. 9.6% males, *p* < 0.001). Among perpetrator categories, “companions” (non‐family members present) were more often implicated in violence against males than females (75.7% vs. 62.2%; *p* = 0.03). At the family level, parental drug abuse (22.6% of male‐child cases vs. 10.2% of female‐child cases; *p* = 0.02) and low overall social level (59.1% vs. 43.9%; *p* = 0.03) were significantly associated with higher rates of violence. Child factors such as hyperactivity (24.9%) and neonatal complications (42.3%) showed no significant associations. Familial risks like parental unemployment (48.4%) and large family size (22.1%) were prevalent but not statistically significant. These findings underscore the role of external social dynamics and parental behavior in exacerbating violence (Table 2).

**Table 2:** Risk factors associated with violence against children with disabilities in the studied groups, Rural Upper Egypt, 2023–2024

Risk factors	*N* = 213		*P*
	**Male N(%)**	**Female (N(%)**	
**Child factors**			
Gender	11 (9.6)	49 (50)	< 0.001*
Hyperactivity	26 (22.6)	27 (27.6)	0.4
Having a learning difficulty	22 (19.1)	18 (18.4)	0.8
Sharing a bed with others	15(13)	11 (11.2)	0.6
Past history of neonatal asphyxia and neonatal intensive care unit admission	51(44.3)	39 (39.8)	0.5
**Perpetrator**			
Parent	66(57.4)	47 (48)	0.1
Brotherhood and sisters	71(61.7)	58 (59.2)	0.7
Companions	87(75.7)	61 (62.2)	0.03*
**Site of violence**			
Outdoor	91(79.1)	84 (85.7)	0.2
Indoor	101 (87.8)	92 (93.9)	0.1
Child's aggressive behavior	69 (60)	53 (54.1)	0.4
Running (Run away from home)	59(51.3)	46 (46.9)	0.5
**Familial factors**			
Low educational level of the parent	62(53.9)	41(41.8)	0.07
Early parenthood < 20 years	9(7.8)	13(13.3)	0.2
Non-employment of the parent	71(61.7)	59 (60.2)	0.8
Divorce or parent separation	12(10.4)	15 (15.3)	0.3
Death of a parent	16(13.9)	12 (12.2)	0.7
Exposure of parents to violence	9(7.8)	6 (6.1)	0.7
Parent conviction	12(10.4)	8 (8.2)	0.6
Parent drug abuse	26(22.6)	10(10.2)	0.02*
Parent history of mental illness	14(12.2)	9 (9.2)	0.5
Increased family size	25(21.7)	21(21.4)	0.9
Low social level	68(59.1)	43(43.9)	0.03*
No social support	36(31.3)	29(29.6)	0.8
Social interaction with caregiver	53(46.1)	40(40.8)	0.4

^*^Significant at *P* value < 0.05

Emotional violence was the most prevalent across all disability types (93.1%–100%), followed by physical (73.6%–78.6%) and sexual violence (21.1%–26.4%). Intellectual disability cases experienced the highest rates of sexual violence (25.9%). No significant differences were observed between disability types, suggesting that emotional abuse is pervasive regardless of disability category, while sexual violence disproportionately affects those with intellectual impairments (Table 3).

**Table 3:** Prevalence of the violence form in relation to the type of disability in the studied groups, Rural Upper Egypt, 2023–2024

Type of disabilities	Physical N(%)	Emotional N(%)	Sexual N(%)
**Physical**			
Male (*n* = 37)	26 (70.3)	35 (94.6)	7 (18.9)
Female (*n* = 35)	27 (77.1)	32 (91.4)	12 (34.3)
Total (*n* = 72)	53 (73.6)	67 (93.1)	19 (26.4)
**Intellectual**			
Male (*n* = 58)	41 (70.7)	55 (94.8)	11 (19)
Female (*n* = 50)	39 (78)	46 (92)	17 (34)
Total (*n* = 108)	80 (74.1)	101 (93.5)	28 (25.9)
**Auditory**			
Male (*n* = 12)	9 (75)	11 (91.7)	2 (16.7)
Female (*n* = 7)	5 (71.4)	6 (85.7)	2 (28.6)
Total (*n* = 19)	14 (73.7)	17 (89.5)	4 (21.1)
**Visual**			
Male (*n* = 8)	6 (75)	8 (100)	1 (12.5)
Female (*n* = 6)	5 (83.3)	6 (100)	2 (33.3)
Total (*n* = 14)	11 (78.6)	14 (100)	3 (21.4)
	0.7	0.8	0.9

Across all physical abuse modalities—such as hitting, throwing objects, biting, hair‐pulling, and forced posturing—the distribution was remarkably consistent: approximately 27% of children experienced one to two episodes, about 35% experienced three to ten episodes, and roughly over 37% of children experienced physical violence more than 10 times, with hitting (37.9%), locking in confined spaces (39.2%), and denial of food (39.1%) being common. No gender disparities were observed. Notably, severe acts like burning (37.5%) and inserting needles (38.2%) were frequently repeated, indicating systemic and prolonged abuse in this cohort (Table 4).

**Table 4:** The frequency of types of physical violence in the studied groups, Rural Upper Egypt, 2023–2024

Types of physical violence	Overall frequency									*P* value
	**1 or 2 times N (%)**			**3–10 times N (%)**			**More than 10 times N (%)**			
	**Male**	**Female**	**Total**	**Male**	**Female**	**Total**	**Male**	**Female**	**Total**	
**Hitting, punching, kicking, or beating a child**	14 (26.9%)	12 (27.9%)	26 (27.4%)	18 (34.6%)	15 (34.9%)	33 (34.7%)	20 (38.5%)	16 (37.2%)	36 (37.9%)	0.9
**Throwing them with something**	6 (26%)	5 (29.4%)	11 (27.5%)	8 (34.8%)	6 (35.3%)	14 (35%)	9 (39.1%)	6 (35.3%)	15 (37.5%)	0.9
**Biting**	8 (26.7%)	6 (27.3%)	14 (26.9%)	11 (36.7%)	8 (36.4%)	19 (36.5%)	11 (36.7%)	8 (36.4%)	19 (36.5%)	0.9
**Pulling or cutting their hair**	6 (25%)	8 (27.6%)	14 (26.4%)	8 (33.3%)	10 (34.5%)	18 (34%)	10 (41.7%)	11 (37.9%)	21 (39.6%)	0.9
**Inserting a needle into their bodies**	5 (26.3%)	4 (26.7%)	9 (26.5%)	7 (36.8%)	5 (33.3%)	12 (35.3%)	7 (36.8%)	6 (40%)	13 (38.2%)	0.9
**Muting their breath with their hand or pillow**	6 (27.3%)	8 (26.7%)	14 (26.9%)	8 (36.4%)	11 (36.7%)	19 (36.5%)	8 (36.4%)	11 (36.7%)	19 (36.5%)	0.9
**Burning by hot objects**	3 (23.1%)	3 (27.3%)	6 (25%)	5 (38.5%)	4 (36.4%)	9 (37.5%)	5 (38.5%)	4 (36.4%)	9 (37.5%)	0.9
**Forcing them to sit or stand in an uncomfortable position**	9 (28.1%)	8 (27.6%)	17 (27.9%)	11 (34.4%)	10 (34.5%)	21 (34.4%)	12 (37.5%)	11 (37.9%)	23 (37.7%)	0.9
**Locking a child up in a small room or tying them with rope or chairs**	11 (26.8%)	9 (27.3%)	20 (27%)	14 (34.1%)	11 (33.3%)	25 (33.8%)	16 (39%)	13 (39.4%)	29 (39.2%)	0.9
**Putting hot chili or bitter food or drink in a child's mouth**	3 (27.3%)	2 (22.2%)	5 (25%)	4 (36.4%)	3 (33.3%)	7 (35%)	4 (36.4%)	4 (44.4%)	8 (40%)	0.9
**Denying a child food for an extended period of time**	10 (26.3%)	8 (25.8%)	18 (26.1%)	13 (34.2%)	11 (35.5%)	24 (34.8%)	15 (39.5%)	12 (38.7%)	27 (39.1%)	0.9
**Forcing a child to carry out hard or difficult work for the benefit of others**	2 (25%)	1 (20%)	3 (23.1%)	3 (37.5%)	2 (40%)	5 (38.5%)	3 (37.5%)	2 (40%)	5 (38.5%)	0.9
**Forcing a child to beg and give the money away**	1 (20%)	1 (33.3%)	2 (25%)	2 (40%)	1 (33.3%)	3 (37.5%)	2 (40%)	1 (33.3%)	3 (37.5%)	0.9

The pattern was uniform across the 24 emotional abuse items: around 23% of participants endured one to two incidents, approximately 33% endured three to ten, and about 44% endured more than ten. Emotional violence was recurrent, with 43%–44% of them subjected to acts such as threats, humiliation, or neglect more than 10 times. Common forms included reproaching for mistakes (43.8%), forced labor (43.3%), and threats of abandonment (44.9%). No gender differences emerged, highlighting the pervasive and chronic nature of psychological abuse across genders (Table 5).

**Table 5:** The frequency of types of emotional violence in the studied groups, Rural Upper Egypt, 2023–2024

Types of emotional violence	Overall frequency									*P* value
	**1 or 2 times N (%)**			**3–10 times N (%)**			**More than 10 times N (%)**			
	**Male**	**Female**	**Total**	**Male**	**Female**	**Total**	**Male**	**Female**	**Total**	
**Asking them to do things without discussion**	14 (23.3%)	12 (23.1%)	26 (23.2%)	20 (33.3%)	17 (32.7%)	37 (33%)	26 (43.3%)	23 (44.2%)	49 (43.8%)	0.99
**Making them feeling estranged and being unfriendly to them**	12 (23.5%)	10 (22.2%)	22 (22.9%)	17 (33.3%)	15 (33.3%)	32 (33.3%)	22 (43.1%)	20 (44.4%)	42 (43.8%)	0.98
**Didn't listen to them when they talk about themselves**	12 (22.2%)	11 (23.9%)	23 (23%)	18 (33.3%)	15 (32.6%)	33 (33%)	24 (44.4%)	20 (43.5%)	44 (44%)	0.98
**Interrupting them for a long period**	12 (24%)	9 (22%)	21 (23.1%)	16 (32%)	14 (34.1%)	30 (33%)	22 (44%)	18 (43.9%)	40 (44%)	0.96
**Insulted, shouted at, ridiculed, shamed and reproaching them for simple mistakes**	13 (22.8%)	11 (22.9%)	24 (22.9%)	19 (33.3%)	16 (33.3%)	35 (33.3%)	25 (43.9%)	21 (43.8%)	46 (43.8%)	1
**Forced to give away money or possessions**	17 (23.6%)	14 (22.6%)	31 (23.1%)	24 (33.3%)	21 (33.9%)	45 (33.6%)	31 (43.1%)	27 (43.5%)	58 (43.3%)	0.99
**Ignored, hidden, or forbidden from participating in social events**	15 (22.7%)	14 (23.7%)	29 (23.2%)	22 (33.3%)	19 (32.2%)	41 (32.8%)	29 (43.9%)	26 (44.1%)	55 (44%)	0.98
**Threatening to send them out of their home**	11 (23.4%)	7 (22.6%)	18 (23.1%)	15 (31.9%)	10 (32.3%)	25 (32.1%)	21 (44.7%)	14 (45.2%)	35 (44.9%)	0.99
**Witnessing of severe beating of a family member or friend**	11 (22.4%)	9 (22.5%)	20 (22.5%)	16 (32.6%)	13 (32.5%)	29 (32.6%)	22 (44.9%)	18 (45%)	40 (44.9%)	1
**Calling them out with undesirable descriptions**	13 (22.8%)	10 (22.2%)	23 (22.5%)	19 (33.3%)	15 (33.3%)	34 (33.3%)	25 (43.9%)	20 (44.4%)	45 (44.1%)	0.99
**Criticizing them for the way they do something**	11 (23.9%)	7 (23.3%)	18 (23.7%)	15 (32.6%)	10 (33.3%)	25 (32.9%)	20 (43.5%)	13 (43.3%)	33 (43.4%)	0.99
**Forcing them to do things they don't like**	13 (22.8%)	10 (23.8%)	23 (23.2%)	19 (33.3%)	14 (33.3%)	33 (33.3%)	25 (43.9%)	18 (42.9%)	43 (43.4%)	0.99
**Comparing them with others to reduce their business**	14 (23.3%)	10 (23.3%)	24 (23.3%)	20 (33.3%)	14 (32.6%)	34 (33%)	26 (43.3%)	19 (44.2%)	45 (43.7%)	0.99
**Depriving them of the thing they like**	13 (23.2%)	11 (23.4%)	24 (23.3%)	18 (32.1%)	15 (31.9%)	33 (32%)	25 (44.6%)	21 (44.7%)	46 (44.7%)	1
**Making them feel the deprivation of parents' emotions towards them**	11 (23.9%)	7 (21.9%)	18 (23.1%)	15 (32.6%)	11 (34.4%)	26 (33.3%)	20 (43.5%)	14 (43.8%)	34 (43.6%)	0.9
**Threatening them with ghosts and evil spirits**	12 (23.5%)	9 (23.1%)	21 (23.3%)	17 (33.3%)	13 (33.3%)	30 (33.3%)	22 (43.1%)	17 (43.6%)	39 (43.3%)	0.99
**Parents do not respond to needs**	13 (23.6%)	10 (23.8%)	23 (23.7%)	18 (32.7%)	14 (33.3%)	32 (33%)	24 (43.6%)	18 (42.9%)	42 (43.3%)	0.99
**No participation in decision-making**	14 (22.2%)	12 (22.6%)	26 (22.4%)	21 (33.3%)	17 (32.1%)	38 (32.8%)	28 (44.4%)	24 (45.3%)	52 (44.8%)	0.99
**Parental preferential prejudice towards sibling(s)**	14 (23%)	11 (23.4%)	25 (23.1%)	20 (32.8%)	16 (34%)	36 (33.3%)	27 (44.3%)	20 (42.6%)	47 (43.5%)	0.98
**Neglected when sick**	11 (22.4%)	8 (22.9%)	19 (22.6%)	16 (32.7%)	12 (34.3%)	28 (33.3%)	22 (44.9%)	15 (42.9%)	37 (44%)	0.98
**Threatened to be hurt or killed**	15 (23.4%)	11 (22.4%)	26 (23%)	21 (32.8%)	16 (32.7%)	37 (32.7%)	28 (43.8%)	22 (44.9%)	50 (44.2%)	0.99
**Told (wish you had never been born/or were dead)**	16 (22.9%)	14 (23.7%)	30 (23.3%)	23 (32.9%)	19 (32.2%)	42 (32.6%)	31 (44.3%)	26 (44.1%)	57 (44.2%)	0.99
**Told they were unloved**	13 (22.8%)	11 (22.9%)	24 (22.9%)	19 (33.3%)	16 (33.3%)	35 (33.3%)	25 (43.9%)	21 (43.8%)	46 (43.8%)	1

Excluding forced circumcision and forced marriage—both reported as singular events (100% in the one‐to‐two incidence category)—other behaviors (e.g., sexual insults, exposure to sexual acts, indecent touching, forced sexual activity) followed a similar pattern to other abuse forms: roughly 20% of participants experienced one to two episodes, 27% experienced three to ten, and about 50% experienced more than ten. Sexual violence was predominantly recurrent (> 10 times: 48.6%–53.8%), particularly forced genital touching (53.8%) and exposure to sexual scenes (50%). Forced circumcision (100%) and marriage (100%) were single-occurrence events. Rates of forced intercourse were 53.3% female vs. 50% males. No statistically significant gender differences were observed for any sexual violence category (Table 6).

**Table 6:** The frequency of types of sexual violence in the studied groups, Rural Upper Egypt, 2023–2024

Types of sexual violence	Overall frequency									*P* value
	**1 or 2 times**			**3–10 times**			**More than 10 times**			
	**Male**	**Female**	**Total**	**Male**	**Female**	**Total**	**Male**	**Female**	**Total**	
**Insulting them with sexual terms**	3 (18.8%)	4 (19%)	7 (18.9%)	4 (25%)	6 (28.6%)	10 (27%)	7 (43.8%)	11 (52.4%)	18 (48.6%)	0.98
**Someone exposes their private parts to the child with disability**	3 (25%)	4 (23.5%)	7 (24.1%)	3 (25%)	5 (29.4%)	8 (27.6%)	6 (50%)	8 (47.1%)	14 (48.3%)	0.96
**Forcing them to see sexual scenes**	3 (21.4%)	4 (20%)	7 (20.6%)	4 (28.6%)	6 (30%)	10 (29.4%)	7 (50%)	10 (50%)	17 (50%)	0.99
**Having their genitals touched or fondled (indecently touched)**	3 (20%)	5 (20.8%)	8 (20.5%)	4 (26.7%)	6 (25%)	10 (25.6%)	8 (53.3%)	13 (54.2%)	21 (53.8%)	0.99
**Forcing them to touch the abuser's genitals**	3 (23.1%)	4 (21.1%)	7 (21.9%)	4 (30.8%)	5 (26.3%)	9 (28.1%)	6 (46.2%)	10 (52.6%)	16 (50%)	0.93
**Trying to embrace and kiss them**	3 (23.1%)	5 (22.7%)	8 (22.9%)	4 (30.8%)	6 (27.3%)	10 (28.6%)	6 (46.2%)	11 (50%)	17 (48.6%)	0.97
**Forcing them to circumcise**	18 (100%)	29 (100%)	47 (100%)	0	0	0	0	0	0	––
**Forcing them into marriage**	6 (100%)	9 (100%)	15 (100%)	0	0	0	0	0	0	––
**Forced to have intercourse**	2 (25%)	3 (20%)	5 (21.7%)	2 (25%)	4 (26.7%)	6 (26.1%)	4 (50%)	8 (53.3%)	12 (52.2%)	0.96

Data are presented as frequency (%)

Mothers (34.8%–41.8%), fathers (35.4%–38.2%), and unrelated participants (44.3%–60%) were frequent perpetrators of physical and sexual violence. Fathers showed significant involvement in sexual violence (*p* = 0.02) as did the “other” perpetrator category (*p* = 0.02), while unrelated participants dominated emotional abuse (56.7%). Strangers and adult neighbors were also prominent, particularly in physical (41.1%) and sexual violence (50.9%). The data emphasize specific risk patterns for familial and non‐familial actors in the context of sexual abuse (Table 7).

**Table 7:** The perpetrator of different types of violence in the studied groups, Rural Upper Egypt, 2023–2024

Perpetrator of different types of violence	Type of violence									*P* value
	**Physical violence (%)**			**Emotional violence N (%)**			**Sexual violence N (%)**			
	**Male (*n* = 82)**	**Female (*n* = 76)**	**Total (*n* = 158)**	**Male (*n* = 110)**	**Female (*n* = 91)**	**Total (*n* = 201)**	**Male (*n* = 21)**	**Female (*n* = 34)**	**Total (*n* = 55)**	
**Mother**	22 (26.8%)	33 (43.4%)	55 (34.8%)	20 (18.2%)	35 (38.5%)	55 (27.4%)	5 (23.8%)	18 (52.9%)	23 (41.8%)	0.3
**Father**	29 (35.4%)	27 (35.5%)	56 (35.4%)	45 (40.9%)	28 (30.8%)	73 (36.3%)	6 (28.6%)	15 (44.1%)	21 (38.2%)	0.02*
**Stepmother**	12 (14.6%)	21 (27.6%)	33 (20.9%)	13 (11.8%)	23 (25.3%)	36 (17.9%)	4 (19%)	13 (38.2%)	17 (30.9%)	0.6
**Stepfather**	3 (3.7%)	10 (13.2%)	13 (8.2%)	4 (3.6%)	3 (3.3%)	7 (3.5%)	1 (4.8%)	9 (26.5%)	10 (18.2%)	0.08
**Sister**	23 (28%)	25 (32.9%)	48 (30.4%)	23 (20.9%)	40 (44%)	63 (31.3%)	2 (9.5%)	10 (29.4%)	12 (21.8%)	0.1
**Brother**	20 (24.4%)	33 (43.4%)	53 (33.5%)	32 (29.1%)	45 (49.5%)	77 (38.3%)	5 (23.8%)	14 (41.2%)	19 (34.5%)	0.47
**Unrelated participants**	31 (37.8%)	39 (51.3%)	70 (44.3%)	67 (60.9%)	47 (51.6%)	114 (56.7%)	14 (66.7%)	19 (55.9%)	33 (60%)	0.08
**Adult neighbor**	40 (48.8%)	25 (32.9%)	65 (41.1%)	48 (43.6%)	43 (47.3%)	91 (45.3%)	12 (57.1%)	16 (47.1%)	28 (50.9%)	0.2
**Teacher**	28 (34.1%)	26 (34.2%)	54 (34.2%)	22 (20%)	21 (23.1%)	43 (21.4%)	3 (14.3%)	6 (17.6%)	9 (16.4%)	0.5
**Stranger**	22 (26.8%)	33 (43.4%)	55 (34.8%)	20 (18.2%)	35 (38.5%)	55 (27.4%)	5 (23.8%)	18 (47.1%)	19 (34.5%)	0.3
**Other**	29 (35.4%)	27 (35.5%)	56 (35.4%)	45 (40.9%)	28 (30.8%)	73 (36.3%)	6 (28.6%)	15 (32.4%)	17 (30.9%)	0.02*

^*^ Significant at *P* value < 0.05

## Discussion

Our study identified several sociodemographic characteristics that create vulnerability contexts for participants with disabilities in rural Upper Egypt. Most children came from families with low socioeconomic status and had caregivers with high illiteracy rates. The gender distribution was relatively balanced, with a slight male predominance. Intellectual disabilities constituted the most common disability type, aligning with findings from Elklit et al. [3] who connected intellectual disabilities with increased victimization risk. Most families in our sample had multiple participants and maintained dual-parent caregiving structures, creating complex caregiving environments that influence vulnerability patterns.

This sociodemographic profile exemplifies what Kyegombe et al. [13] describe as the "intersectionality of disadvantage" where disability, poverty, and limited education collectively heighten vulnerability to violence. The high proportion of firstborn children with disabilities suggests challenges in early parenting experiences, a factor that Cuartas et al. [14] emphasize as critical in violence prevention. These findings reinforce Banks et al. [15] observation that child protection mechanisms in resource-limited settings often remain underdeveloped and particularly non-inclusive of disability needs.

Our results revealed significant gender disparities in violence exposure, with females experiencing higher rates than males. This contrasts with Elklit et al. [3], who found boys with disabilities were more frequently victimized in criminal contexts, suggesting context-dependent gender dimensions in violence patterns. We found significant associations between parental drug abuse and violence in the male cohort, paralleling Elklit et al. [3] identification of parental characteristics as key risk multipliers. The relationship between low social status and increased violence aligns with Fang et al.’s meta-analysis [16] highlighting heightened vulnerability in economically disadvantaged contexts.

Unlike Koivula et al. [17], who identified neurological or psychological conditions as particularly associated with maternal psychological aggression, our study found no significant associations with child factors such as hyperactivity or neonatal complications. The role of non-family "companions" in perpetrating violence against male participants highlights the external social risks identified by Njelesani et al. [18], who noted that community participation patterns shape violence exposure.

Our study revealed remarkably consistent patterns of violence across disability categories, with emotional violence being nearly universal, followed by physical and sexual violence. This pattern aligns with Fang et al. [16] global meta-analysis reporting substantial violence prevalence among participants with disabilities. Unlike Elklit et al. [3] who identified varying risk levels by disability type, our study found no significant differences between disability categories, suggesting what Njelesani [19] describes as "disability-based violence" where the presence of any disability, rather than the specific type, becomes the primary vulnerability factor.

However, our finding that intellectual disability cases experienced the highest rates of sexual violence corresponds with Elklit et al. [3] conclusion regarding heightened sexual victimization risk in this population. These findings are supported by Hillis et al. [2] analysis showing that participants with disabilities are significantly more likely to experience violence overall, with varying odds ratios for physical and sexual violence. The pervasiveness of emotional violence across all disability types indicates a concerning normalization of psychological maltreatment, as identified by Banks et al. [15] in their qualitative exploration of violence experiences.

Our analysis revealed disturbing patterns of repetitive physical abuse, with many participants experiencing severe physical violence multiple times. The distribution pattern shows approximately equal proportions experiencing few, moderate, or numerous episodes, suggesting established patterns of recurring abuse. This chronic exposure aligns with Seppälä et al. [20] findings that participants with multiple disabilities face a substantially higher risk of serious violence compared to non-disabled peers.

The prevalence of particularly harmful acts like burning and inserting needles indicates what Omer et al. [12] described as severe physical maltreatment. The absence of significant gender differences in physical violence frequency diverges somewhat from broader patterns noted by Elklit et al. [3]. The high frequency of confinement practices and food denial represents the intersection of physical punishment and neglect described by Njelesani et al. [18] in West African contexts. This combination of frequency and severity suggests deeply entrenched patterns of physical abuse requiring targeted intervention, aligning with Pundir et al. [21] emphasis on contextually appropriate violence reduction strategies.

Emotional violence in our study demonstrated a pervasive and recurring nature, with a substantial proportion of participants experiencing numerous episodes of various forms of emotional abuse. This finding aligns with Banks et al. [15], who identified verbal abuse as among the most common forms of violence experienced by participants with disabilities. The recurrent nature of specific emotional violence types—including reproaching for mistakes, forced labor, and threats of abandonment—reveals deeply embedded patterns of psychological maltreatment. These patterns correspond with Koivula et al. [17] observation that mothers of participants with disabilities reported significantly higher levels of psychological aggression. The absence of gender differences in emotional violence frequency indicates the universality of this abuse form, consistent with Njelesani et al. [22] findings that stigmatizing beliefs about disability, rather than gender, often drive emotional maltreatment. The frequency distribution pattern suggests systematic, recurring emotional abuse rather than isolated incidents, corresponding with Devries et al. [23] observations of high baseline rates of violence in school settings for students with disabilities.

Our findings revealed concerning patterns of sexual violence, with approximately half of the affected participants experiencing numerous episodes. This chronic exposure aligns with Hillis et al. meta-analysis [2] identifying a substantial prevalence of sexual violence against participants with disabilities and a significantly elevated risk compared to non-disabled peers. The high recurrence of forced genital touching and exposure to sexual scenes indicates systematic sexual abuse patterns, corresponding with Elklit et al. [3] findings of heightened sexual victimization risk, particularly among participants with intellectual disabilities.

Certain forms of sexual violence—forced circumcision and forced marriage—were experienced as singular events, representing distinct cultural practices with long-term consequences. The similar rates of forced intercourse between genders contrast with common assumptions about gender-based patterns in sexual violence, suggesting what Fang et al. [16] described as complex intersections between disability status and other vulnerability factors. The distribution pattern across sexual violence forms indicates recurring victimization rather than isolated incidents, supporting Kyegombe et al. [13] emphasis on specialized protection mechanisms for participants with disabilities.

Our analysis revealed complex perpetrator patterns with both familial and non-familial actors significantly involved. Mothers, fathers, and unrelated participants emerged as frequent perpetrators, with specific patterns across violence types. The significant involvement of fathers in sexual violence represents a particularly concerning finding, adding nuance to Koivula et al. [17] focus on maternal aggression.

The prominence of strangers and adult neighbors, particularly in physical and sexual violence, corresponds with Njelesani et al. [18] identification of community-level perpetrators. The dominance of unrelated participants in emotional abuse aligns with Njelesani et al. [24] observations of peer-perpetrated violence against students with disabilities in Zambian schools. The significant involvement of "other" perpetrator categories in sexual violence suggests what Kyegombe et al. [13] describe as the complex web of potential perpetrators that child protection systems must address.

These diverse perpetrator patterns are supported by Banks et al. [15] conclusion that protection mechanisms must operate at multiple levels to effectively safeguard participants with disabilities. The high involvement of both family members and unrelated individuals in all forms of violence underscores Cuartas et al. [14] emphasis on multi-component interventions addressing both familial and community factors to prevent violence against vulnerable participants.

### Limitations of the study

This study has several limitations that may influence the interpretation of its findings. The retrospective design and reliance on self-reported data introduce potential recall and social desirability biases, as participants may not accurately remember past events or may alter their responses to align with social expectations. The sample, drawn from two rural villages, limits the generalizability of the results to broader populations. Verification of disability through official documentation may exclude individuals without formal records, leading to an underestimation of disability prevalence, especially in rural or low-resource settings where bureaucratic barriers, stigma, or poverty hinder access to such documentation. The cross-sectional approach precludes causal inferences, making it impossible to determine the directionality of associations between variables. Additionally, potential underreporting of sensitive issues and focus on specific disabilities could influence prevalence estimates, as individuals may be reluctant to disclose experiences of violence, and the study's focus may not encompass all types of disabilities. These factors should be considered when interpreting findings and designing future research.

## Conclusions

The study reveals high rates of emotional, physical, and sexual violence against participants with disabilities in rural Upper Egypt, particularly affecting females and those with intellectual disabilities. Key risk factors include low socioeconomic status, parental drug abuse, and non-familial perpetrators. Findings underscore the urgent need for multi-level interventions, including strengthening legal enforcement, community education, and family support systems. Addressing the intersection of poverty, disability stigma, and systemic neglect is critical to safeguarding this vulnerable population and aligning with global child protection frameworks.

## Data Availability

The datasets generated and/or analyzed during the current study are available from the corresponding author on reasonable request.

## Abbreviations

ISPCAN: International Society for the Prevention of Child Abuse and Neglect
ICAST-R: ISPCAN Child Abuse Screening Tool – Retrospective version.
WHO: World Health Organization
CDC: Disease Control and Prevention
